# The impact of COVID-19 on participation in Australia’s National Bowel Cancer Screening Program by people with severe mental illness: A national data linkage study

**DOI:** 10.1177/00048674251336034

**Published:** 2025-04-28

**Authors:** Claudia Bull, Katrina Spilsbury, David Lawrence, Karinna I. Saxby, Steve Kisely

**Affiliations:** 1 Princess Alexandra Hospital Southside Clinical Unit, Greater Brisbane Clinical School, Medical School, Faculty of Health, Medicine and Behavioural Sciences, The University of Queensland, Woolloongabba, QLD, Australia; 2 Queensland Centre for Mental Health Research, Faculty of Health, Medicine and Behavioural Sciences, The University of Queensland, Brisbane, QLD, Australia; 3 Curtin School of Population Health, Faculty of Health Sciences, Curtin University, Perth, WA, Australia; 4 Institute for Health Research, The University of Notre Dame Australia, Fremantle, WA, Australia; 5 Melbourne Institute: Applied Economic & Social Research, Faculty of Business and Economics, The University of Melbourne, Melbourne, VIC, Australia; 6 Metro South Addiction and Mental Health Service, Brisbane, QLD, Australia

**Keywords:** National Bowel Cancer Screening Program, COVID-19, Australia, data linkage, severe mental illness, colonoscopy

## Abstract

**Objective:**

The impact of COVID-19 on Australia’s National Bowel Cancer Screening Program remains unclear, especially for individuals with severe mental illness. These individuals have historically participated in the National Bowel Cancer Screening Program at significantly lower rates than the general population. This study aimed to understand the impact of COVID-19 on participation in Australia’s National Bowel Cancer Screening Program among individuals with severe mental illness.

**Methods:**

Cohort study using deidentified linked health and National Bowel Cancer Screening Program data. We compared participation in the National Bowel Cancer Screening Program between individuals with and without severe mental illness by examining rates of participation (returning an immunochemical faecal occult blood test), returning a valid immunochemical faecal occult blood test, receiving a positive immunochemical faecal occult blood test result and undergoing a follow-up colonoscopy before (25 January 2018–24 January 2020) and during (25 January 2020–31 July 2021) the COVID-19 pandemic.

**Results:**

Overall National Bowel Cancer Screening Program participation fell by 10.3% from pre-COVID to during COVID. Less than one-quarter (23.9%) of people with severe mental illness participated in the National Bowel Cancer Screening Program during the COVID-19 pandemic compared to 30.5% before. People with severe mental illness were less likely to return a valid immunochemical faecal occult blood test and more likely to return a positive immunochemical faecal occult blood test result both before and during the pandemic, compared to the general population. They were also significantly less likely to have a colonoscopy following positive immunochemical faecal occult blood test result (pre-COVID adjusted relative risk = 0.97, 95% confidence interval: 0.94–1.01, vs during COVID adjusted relative risk = 0.87, 95% CI: 0.82–0.91).

**Conclusion:**

The pandemic significantly reduced the rate at which all Australians participated in the National Bowel Cancer Screening Program. Disparities between people with severe mental illness and the general population generally improved with the exception of follow-up colonoscopy after positive immunochemical faecal occult blood test result.

## Introduction

Australia’s National Bowel Cancer Screening Program (NBCSP) provides biennial primary screening for colorectal cancer (CRC). Since 2006, Australians aged 50–74 years have been invited to participate in the NBCSP using an immunochemical faecal occult blood test (iFOBT) via the national postal service (Nickson et al., 2023). These self-administered home tests can detect microscopic amounts of blood in a stool sample which may indicate abnormality (Australian Institute of Health and Welfare, 2021). If a valid and positive result is obtained (indicating abnormality), a follow-up colonoscopy is recommended within 120 days. Similar screening programs with varying participation rates exist in Spain, The Netherlands, Ireland, Italy, Croatia, Lithuania, Slovenia, the United Kingdom, Czech Republic, France, the United States, Canada, Chile, Japan, South Korea, Taiwan and Thailand (Navarro et al., 2017). Results from meta-analyses show that routine screening using iFOBT between the ages of 45 and 80 years can reduce CRC mortality by up to 69%, with earlier detection leading to better survival (Zheng et al., 2023).

### Hypothesised and realised disruptions to the NBCSP during COVID-19

Soon after the World Health Organization declared COVID-19 to be a global pandemic in March 2020, the Cancer Council of Australia released a report modelling hypothetical disruptions to the NBCSP with the imposition of state and national infection control measures (Worthington et al., 2020). Results suggested that any disruption to the program would lead to delayed CRC detection in the short term, as well as upstaged (i.e. more progressed) CRC cases, and higher numbers of new cases and deaths in the long term (Worthington et al., 2020). A separate study commissioned by the Australian Government Department of Health hypothesised that a 3-month disruption to the NBCSP would reduce colonoscopy rates by 9.8%, reduce rates of CRC diagnosis by 2.8% and increase the detection of CRC at stages 3–4 by 1% (Nickson et al., 2023). The consequences of a 12-month disruption were hypothesised to be more drastic; colonoscopy rates would reduce by 39.0%, rates of CRC diagnosis would reduce by 12.1% and the detection of CRC at stages 3–4 would increase by 7% (Nickson et al., 2023).

However, the reality of COVID-19 disruptions to the NBCSP remain ambiguous. Evidence shows that the relative rate of nation-wide participation in the NBCSP reduced by 6% between 2018–2019 and 2020–2021 (Irwin et al., 2024). Yet, the number of colonoscopies occurring within the recommended timeframe of 120 days increased by 14.5% (Irwin et al., 2024). This could potentially be attributable to selection of ‘more engaged’ health consumers among those who continued to participate in NBCSP during the pandemic. To this end, cohort analyses are essential to disentangle the true extent to which the pandemic impacted NBCSP participation across different population subgroups.

### NBCSP participation among people with severe mental illness

A particularly important cohort to study in this context is people with severe mental illness (SMI). Individuals with SMI – including schizophrenia, bipolar and major depressive disorder – already experience a lower life expectancy of approximately 15 years compared to the general population (Chan et al., 2023). There is also evidence that they are significantly less likely to participate in the NBCSP by returning a iFOBT (adjusted incidence rate ratio [IRR_adj_]= 0.70, 95% confidence interval [CI]: 0.69–0.70), and are significantly less likely to have a colonoscopy following a positive iFOBT (IRR_adj_ = 0.88, 95% CI: 0.85–0.92) (Kisely et al., 2024). Risk factors such as amotivation, cognitive impairments, living remotely, socioeconomic disadvantage and returning invalid tests further compound low rates of participation among people with SMI, thereby increasing the likelihood that people with SMI are diagnosed with CRC at a later, less treatable stage (Kisely et al., 2024; Seppänen et al., 2024).

Thus, the purpose of this study was to understand the impact COVID-19 had on participation in Australia’s NBCSP among individuals with and without SMI. Specifically, we sought to compare participation in the NBCSP between individuals with SMI to the general population by examining rates of returning an iFOBT, returning a valid iFOBT, receiving a positive iFOBT result, and undergoing a follow-up colonoscopy before and during the COVID-19 pandemic. Our previous research was confined to NBCSP participation in people with SMI following their first invitation in the years prior to the pandemic. This study considered participation both before and during the pandemic irrespective of whether this was following a first or subsequent NBCSP invitation.

## Methods

### Study design

This investigation is part of the Colorectal cancer Outcomes in people with Severe Mental Illness Cohort (COSMIC) Study (Protani et al., 2021). COSMIC is a representative population-based cohort study that uses de-identified, linked administrative health data. As this study focused on comparing participation in the NBCSP between individuals with and without SMI before and during the COVID-19 pandemic, we used NBCSP data from 25 January 2018 to 24 January 2020 to represent the pre-COVID period, 25 January 2020 to 31 July 2021 for the during COVID period. The first case of COVID-19 in Australia was confirmed on 25 January 2020, and 31 July 2021 was the latest date of NBCSP invitation available in the COSMIC dataset.

The authors assert that all procedures contributing to this work comply with the ethical standards of the relevant national and institutional committees on human experimentation and with the Helsinki Declaration of 1975, as revised in 2013. All procedures involving human subjects/patients were approved by The University of Queensland Human Research Ethics Committee (2019000296) and the Australian Institute of Health and Welfare Ethics Committee (E2019-5-1108). A pre-study protocol was published (Protani et al., 2021) and we prospectively registered the project with the Australian and New Zealand Clinical Trials Registry (Trial no: ACTRN12620000781943) (Protani et al., 2021). Strengthening the Reporting of Observational Studies in Epidemiology (STROBE) guidance was adhered to (von Elm et al., 2008) (Supplementary Material).

### Data sources

The COSMIC dataset comprises six Commonwealth data sources, including the Pharmaceutical Benefits Scheme (PBS), Medicare Enrollment File (MEF), Australian Cancer Database (ACD), NBCSP, Medicare Benefits Schedule (MBS) and National Death Index (NDI). The PBS comprises data on the use of all prescription medications, including those subsidised by the Australian government, other than medications dispensed in-hospital. The MEF comprises all Australian citizens and permanent residents enrolled to receive Medicare benefits (Australia’s universal health insurance scheme). The ACD comprises all new cases of malignant cancer diagnosed in Australia (excluding basal and squamous cell carcinomas). The NBCSP comprises all NBCSP invitations, completed iFOBT screening and results, follow-up colonoscopy invitations, completed follow-up colonoscopies and follow-up colonoscopy results. The MBS comprises all Medicare-subsidised health service utilisation by eligible Australians, including iFOBT use, sigmoidoscopies and colonoscopies that may not have been recorded in the NBCSP. Further details about the COSMIC dataset can be found in the published protocol (Protani et al., 2021).

### Cohort derivation

The SMI and comparison cohorts were identified based on PBS medication dispensing histories. A person was considered eligible for inclusion in the SMI cohort after being dispensed two or more prescriptions within a 12-month period for atypical antipsychotics with an indication-specific authority code for schizophrenia or bipolar affective disorder, or lithium (Anatomical Therapeutic Chemical [ATC] codes N05AH, N05AX, N05AE, N05AL and N05AN). Persons with a history of low-dose SMI medication use only (e.g. Quetiapine < 100 mg) were excluded.

The control cohort was identified from the MEF, which comprised all people eligible for Medicare benefits (>80% of Australians) (Harris et al., 2019). For every one SMI cohort member, nine Australians of the same age range who had never been prescribed any psychotropic medications (antipsychotics, lithium or antidepressants [ATC N06A]) were randomly selected as the general population group. Individuals who were prescribed antidepressants were deliberately excluded from the control cohort because people with depression and anxiety were considered to have a different NBCSP participation profile compared to the general population.

After defining the SMI and control cohorts, all people who were not invited to participate in the NBCSP between 25 January 2018 and 31 July 2021 were subsequently excluded. The NBCSP currently offers biennial screening for all eligible Australians aged between 50 and 74 years, unless they previously opted out of the program or previously had a positive iFOBT result confirmed by colonoscopy (in which case they may have received follow-up management with their General Practitioner). Participation is via a posted iFOBT screening kit.

### Outcome measures

The outcomes of interest for this study were participating in the NBCSP (evidenced by returning an iFOBT), returning a valid iFOBT, receiving positive iFOBT results and undergoing follow-up colonoscopy after positive iFOBT, before and during the COVID-19 pandemic. These outcomes are contingent on one another. That is, a decrease in the number of positive iFOBT results will lead to fewer people being invited for follow-up colonoscopies. Fewer iFOBT tests returned will likely lead to fewer positive results. Specifically, we investigated whether these outcomes differed according to SMI or general population groups. Colonoscopies recorded in the MBS data (service item numbers 32084, 32087-32090, 32093, 32222-32229) that followed a positive iFOBT result and occurred before and during the COVID-19 pandemic were identified and included. As cancer records were only available up to 31 December 2017, we were unable to examine the type of cancer developed nor spread.

We also examined whether participating in the NBCSP (evidenced by returning an iFOBT), returning a valid iFOBT, receiving positive iFOBT results, and undergoing follow-up colonoscopy after positive iFOBT differed depending on COVID-19 lockdown severity. Based on information from the Australian Bureau of Statistics (ABS) (ABS, 2021), we classified Australian states and territories into the following lockdown severity groupings: low lockdown severity = Northern Territory, Queensland, South Australia, Tasmania and Western Australia; moderate lockdown severity = New South Wales and Australian Capital Territory; high lockdown severity = Victoria.

### Statistical analysis

We examined the proportion of individuals who participated in the NBCSP (by returning an iFOBT), returning a valid iFOBT, returning a positive iFOBT and having a colonoscopy following positive iFOBT both before and during the COVID-19 pandemic. We also described the sociodemographic data for control and SMI cohorts before and during the COVID-19 pandemic using means, standard deviations, and proportions. Mean differences (MD) and unadjusted relative risk (RR) ratios with 95% CIs were calculated to compare control and SMI cohorts.

We constructed log-binomial regression models to estimate unadjusted and adjusted RR ratios (with 95%CI) showing the differences between control and SMI cohorts, before and during the COVID-19 pandemic, for each of the abovementioned outcomes. The following variables were used in adjustment: age at NBCSP invitation, sex (male, female), level of remoteness (major city, inner regional, outer regional and remote or very remote) and index of socioeconomic disadvantage (in quintiles) (ABS, 2018). We have previously identified these to be confounding variables (Kisely et al., 2024). We were unable to include First Nations’ status in adjustment due to high levels of missing data (>40%). We also examined differences between control and SMI cohorts, before and during the COVID-19 pandemic for the abovementioned outcomes relative to whether participants lived in low, moderate or high COVID-19 lockdown severity states/territories. All analyses were undertaken in SAS (version 9.4, 2023).

## Results

Table 1 compares the sociodemographic characteristics of the SMI and control cohorts before and during the COVID-19 pandemic. Across both time periods, individuals in the SMI cohort were younger, more likely to be female and more likely to live in inner or outer regional areas. The SMI cohort was also significantly more likely to be represented in the bottom two quintiles of socioeconomic disadvantage. One notable difference is that the SMI cohort were significantly less likely to have received their first invitation during the pandemic compared to people without SMI (RR = 0.67, 95% CI: 0.66–0.69), but were more likely to have received their second, third, fourth or higher invitation.

**Table 1.** Comparing the sociodemographic characteristics of the SMI and control cohorts before and during the COVID-19 pandemic.

Baseline characteristics	Before the COVID-19 pandemic *n* = 985,227			During the COVID-19 pandemic *n* = 937,887		
	SMI cohort *n* = 120,651	Control cohort *n* = 864,576	MD (95%CI) // Unadjusted RR (95% CI)	SMI cohort *n* = 115,655	Control cohort *n* = 822,232	MD (95%CI) // Unadjusted RR (95% CI)
Average age (mean ± SD)	60.4 ± 7.1	61.1 ± 7.2	0.70 (0.66–0.74)	59.9 ± 7.0	60.3 ± 7.3	0.40 (0.36–0.44)
Sex						
Male	57,494 (47.6%)	474,056 (54.8%)	0.87 (0.86–0.87)	57,833 (50.0%)	443,423 (53.9%)	0.93 (0.92–0.93)
Female	63,157 (52.4%)	390,520 (45.2%)	1.16 (1.15–1.17)	57,822 (50.0%)	378,809 (46.1%)	1.09 (1.08–1.09)
Indigenous status^a^						
Not First Nations	69,132 (57.3%)	556,944 (64.4%)	0.98 (0.97–0.98)	62,191 (53.8%)	482,268 (58.7%)	0.97 (0.97–0.97)
Identified as First Nations	2913 (2.4%)	9078 (1.0%)	2.52 (2.42–2.63)	3077 (2.7%)	9549 (1.2%)	2.43 (2.33–2.53)
State of residence^b^						
Australian Capital Territory	1624 (1.3%)	14,560 (1.7%)	0.80 (0.76–0.84)	1442 (1.2%)	13,208 (1.6%)	0.78 (0.73–0.82)
New South Wales	40,058 (33.2%)	288,291 (33.3%)	1.00 (0.99–1.00)	38,214 (33.0%)	274,059 (33.3%)	0.99 (0.98–1.00)
Northern Territory	538 (0.4%)	7990 (0.9%)	0.48 (0.44–0.53)	626 (0.5%)	9715 (1.2%)	0.46 (0.42–0.50)
Queensland	23,604 (19.6%)	166,543 (19.3%)	1.02 (1.00–1.03)	22,110 (19.1%)	153,242 (18.6%)	1.03 (1.01–1.04)
South Australia	9949 (8.2%)	61,926 (7.2%)	1.15 (1.13–1.17)	9239 (8.0%)	56,587 (6.9%)	1.16 (1.14–1.19)
Tasmania	2747 (2.3%)	18,458 (2.1%)	1.07 (1.02–1.11)	2893 (2.5%)	17,847 (2.2%)	1.15 (1.11–1.20)
Victoria	31,690 (26.3%)	216,322 (25.0%)	1.05 (1.04–1.06)	31,308 (27.1%)	212,232 (25.8%)	1.05 (1.04–1.06)
Western Australia	10,339 (8.6%)	89,618 (10.4%)	0.83 (0.81–0.84)	9725 (8.0%)	84,402 (10.3%)	0.82 (0.80–0.84)
Level of remoteness						
Major cities	79,298 (65.7%)	603,880 (69.8%)	0.94 (0.94–0.95)	75,385 (65.2%)	582,272 (70.8%)	0.92 (0.92–0.92)
Inner regional	29,797 (24.7%)	175,224 (20.3%)	1.22 (1.21–1.23)	28,988 (25.1%)	160,015 (19.5%)	1.29 (1.27–1.30)
Outer regional	10,265 (8.5%)	72,461 (8.4%)	1.02 (1.00–1.04)	9904 (8.6%)	66,199 (8.1%)	1.06 (1.04–1.09)
Remote or very remote	1291 (1.1%)	13,011 (1.5%)	0.71 (0.67–0.75)	1378 (1.2%)	13,746 (1.7%)	0.71 (0.67–0.75)
Index of socioeconomic disadvantage^c^						
Highest (most advantaged)	21,800 (18.1%)	234,508 (27.1%)	0.67 (0.66–0.67)	19,889 (17.2%)	224,010 (27.2%)	0.63 (0.62–0.64)
4th quintile	20,806 (17.2%)	173,869 (20.1%)	0.86 (0.85–0.87)	19,870 (17.2%)	166,935 (20.3%)	0.85 (0.84–0.86)
3rd quintile	24,056 (19.9%)	167,830 (19.4%)	1.03 (1.01–1.04)	23,301 (20.1%)	158,953 (19.3%)	1.04 (1.03–1.06)
2nd quintile	23,471 (19.4%)	145,109 (16.8%)	1.16 (1.14–1.17)	22,805 (19.7%)	135,216 (16.4%)	1.20 (1.18–1.21)
Lowest (most disadvantage)	26,697 (22.1%)	132,964 (15.4%)	1.44 (1.42–1.46)	26,050 (22.5%)	126,119 (15.3%)	1.47 (1.45–1.49)
Year of NBCSP invitation						
2018	56,307 (46.7%)	416,080 (48.1%)	0.97 (0.96–0.98)	–	–	–
2019	63,906 (53.0%)	444,013 (51.4%)	1.03 (1.03–1.04)	–	–	–
2020	438 (0.3%)	4483 (0.5%)	0.70 (0.63–0.77)	66,119 (57.2%)	493,357 (60.0%)	0.95 (0.95–0.96)
2021 (⩽31 July)	–	–	–	49,536 (42.8%)	328,875 (40.0%)	1.07 (1.06–1.08)
*n*th invitation to participate in the NBCSP						
1st invitation	15,107 (12.5%)	109,397 (12.7%)	0.99 (0.97–1.01)	11,797 (10.2%)	124,617 (15.2%)	0.67 (0.66–0.69)
2nd invitation	32,646 (27.1%)	233,927 (27.1%)	1.00 (0.99–1.01)	21,962 (19.0%)	143,839 (17.5%)	1.09 (1.07–1.10)
3rd invitation	61,447 (50.9%)	439,483 (50.8%)	1.00 (1.00–1.01)	35,146 (30.4%)	237,466 (28.9%)	1.05 (1.04–1.06)
⩾4th invitation	11,451 (9.5%)	81,769 (9.5%)	1.00 (0.99–1.02)	46,732 (40.4%)	316,310 (38.5%)	1.05 (1.04–1.06)

NBCSP: National Bowel Cancer Screening Program; SD: standard deviation; MD: mean difference; CI: confidence interval; RR: relative risk; SMI: severe mental illness.

a Missing data for *n* = 347,160 (35.2%) and *n* = 380,802 (40.6%) of before and during COVID samples, respectively.

b Missing data for *n* = 970 (0.1%) and *n* = 1038 (0.1%) of before and during COVID samples, respectively.

c Missing data for *n* = 14,117 (1.4%) and *n* = 14,739 (1.6%) of before and during COVID samples, respectively.

**Figure 1.**
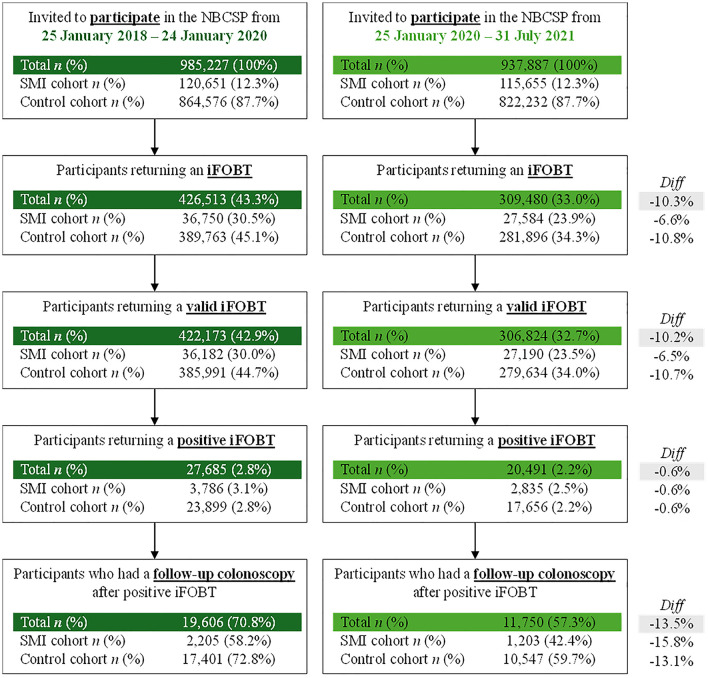
Participation in the NBCSP before (left) and during (right) the COVID-19 pandemic. The denominator for participants returning an iFOBT, returning a valid iFOBT and returning a positive iFOBT are the total numbers as represented in the box describing the number invited to participate in the NBCSP. The denominator for participants who had a follow-up colonoscopy is represented in the box describing returning a positive iFOBT.

Figure 1 shows the number and proportion of individuals in the control and SMI cohorts participating in the NBCSP before and during the COVID-19 pandemic. Overall, there was a substantial drop in participation (defined as returning an iFOBT) from before COVID (43.3%) to during COVID (33.0%). Less than one-quarter of SMI cohort members returned an iFOBT test during COVID (23.9%). That lead to lower rates of returning valid iFOBTs, receiving positive iFOBT results, and having follow-up colonoscopies during the COVID-19 pandemic. Despite an overall reduction in NBCSP participation of 10.3%, the reduction in follow-up colonoscopies was more marked at 13.5%. Individuals in the SMI cohort were disproportionately affected. Where 58.32% of the SMI cohort had a follow-up colonoscopy after positive iFOBT before COVID, this reduced to 42.4% during COVID.

**Figure 2.**
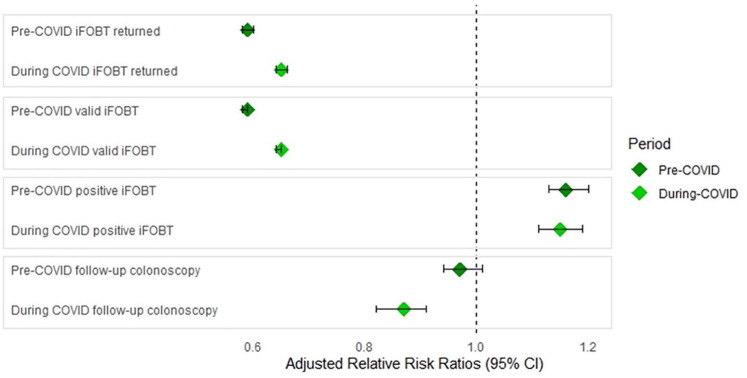
Adjusted relative risk ratios pre- and during COVID relative to specific invitation, participation and follow-up stages of the NBCSP.

Table 2 shows the difference between SMI and control cohorts at invitation, participation and follow-up stages of the NBCSP before and during the COVID-19 pandemic. Compared to pre-COVID conditions, the SMI cohorts’ risk of returning an iFOBT during COVID significantly increased from RR_adj_ = 0.59 (95% CI: 0.58–0.60) to RR_adj_ = 0.65 (95% CI: 0.64–0.66) relative to controls, and receiving a positive iFOBT result reduced slightly from RR_adj_ = 1.16 (95% CI: 1.13–1.20) to RR_adj_ = 1.15 (95% CI: 1.11–1.19). There was also a significant reduction in the number of SMI cohort members undergoing follow-up colonoscopies (after positive iFOBT) before (RR_adj_ = 0.97, 95% CI: 0.94–1.01) and during (RR_adj_ = 0.87, 95% CI: 0.82–0.91) the COVID-19 pandemic. Figure 2 shows the differences in adjusted RR ratios before and during the COVID-19 pandemic for these outcomes.

**Table 2.** Differences between SMI and control cohorts at specific invitation, participation and follow-up stages of the NBCSP before and during the COVID-19 pandemic.

NBCSP invitation, participation and follow-up stage	Before the COVID-19 pandemic				During the COVID-19 pandemic			
	SMI cohort	Control cohort	Unadjusted RR (95% CI)	Adjusted RR (95% CI)^a^	SMI cohort	Control cohort	Unadjusted RR (95% CI)	Adjusted RR (95% CI)^a^
Invited to participate in NBCSP	120,651 (100%)	864,576 (100%)	–	–	115,655 (100%)	822,232 (100%)	–	–
iFOBT returned	36,750 (30.5%)	389,763 (45.1%)	0.68 (0.67–0.68)	0.59 (0.58–0.60)	27,584 (23.9%)	281,896 (34.3%)	0.70 (0.69–0.70)	0.65 (0.64–0.66)
Valid iFOBT	36,182 (30.0%)	385,991 (44.6%)	0.67 (0.67–0.68)	0.59 (0.58–0.59)	27,190 (23.5%)	279,634 (34.0%)	0.69 (0.68–0.70)	0.65 (0.64–0.65)
Positive iFOBT	3786 (3.1%)	23,899 (2.8%)	1.14 (1.10–1.17)	1.16 (1.13–1.20)	2835 (2.5%)	17,656 (2.2%)	1.14 (1.10–1.19)	1.15 (1.11–1.19)
Follow-up colonoscopy (after a positive iFOBT)	2205 (58.2%)	17,401 (72.8%)	0.91 (0.87–0.95)	0.97 (0.94–1.01)	1203 (42.3%)	10,547 (59.7%)	0.81 (0.76–0.86)	0.87 (0.82–0.91)

NBCSP: National Bowel Cancer Screening Program; NBCSP: National Bowel Cancer Screening Program; SMI: severe mental illness; IRR: incidence rate ratio; iFOBT: immuochemical faecal occult blood test.

The denominator for participants returning an iFOBT, returning a valid iFOBT and returning a positive iFOBT are the total numbers as represented by the row describing the number invited to participate in the NBCSP. The denominator for participants who had a follow-up colonoscopy is represented by the row describing returning a positive iFOBT.

a Adjusted for participant age, level of remoteness, index of socioeconomic disadvantage and sex.

**Figure 3.**
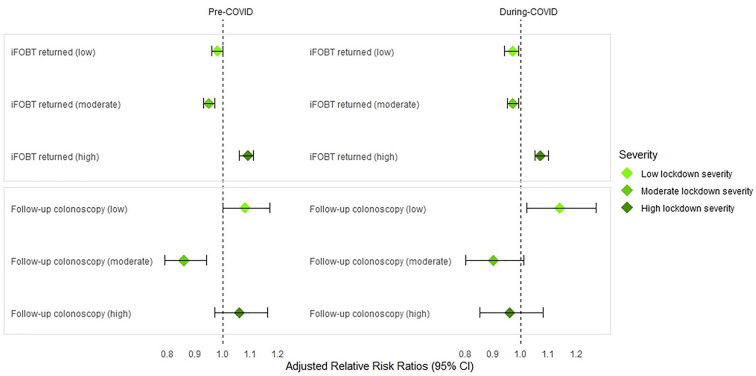
Participation rates and follow-up colonoscopies (after positive iFOBT) between individuals with SMI and controls, before and during COVID-19, relative to low, moderate and high lockdown severity regions.

Table 3 shows the difference between SMI and control cohorts at invitation, participation and follow-up stages of the NBCSP before and during the COVID-19 pandemic, relative to lockdown severity. During COVID, people with SMI living in high lockdown severity areas had significantly higher risk of participating in the NBCSP by returning an iFOBT compared to controls and compared to people with SMI living in low and moderate lockdown severity areas. This same pattern was evident pre-COVID and did not differ significantly between pre- and during COVID periods (RR_adj_ = 1.07, 95% CI: 1.05–1.10 vs RR_adj_ = 1.09, 95% CI: 1.06–1.11, respectively). People with SMI living in low lockdown severity areas had significantly higher risk of undergoing a follow-up colonoscopy (after positive iFOBT) during COVID compared to controls (RR_adj_ = 1.14, 95% CI: 1.02–1.27). This did not differ significantly to pre-COVID (RR_adj_ = 1.08, 95% CI: 1.01–1.11). Figure 3 shows these differences between individuals with SMI and controls, before and during COVID-19 relative to lockdown severity.

**Table 3.** Differences between SMI and control cohorts at specific invitation, participation and follow-up stages of the NBCSP before and during the COVID-19 pandemic across high, moderate and low regions of lockdown severity.

NBCSP participation and follow-up stage, by region of lockdown severity^a,b^	Before the COVID-19 pandemic				During the COVID-19 pandemic			
	SMI cohort	Control cohort	Unadjusted RR (95% CI)	Adjusted RR (95% CI)^c^	SMI cohort	Control cohort	Unadjusted RR (95% CI)	Adjusted RR (95% CI)^c^
Invited to participate in NBCSP								
Low lockdown severity	47,177 (39.1%)	344,535 (40.0%)	0.98 (0.97–0.99)	0.97 (0.96–0.98)	44,593 (38.6%)	321,793 (39.2%)	0.99 (0.98–0.99)	0.97 (0.96–0.98)
Moderate lockdown severity	41,682 (34.6%)	302,851 (35.0%)	0.99 (0.98–0.99)	0.97 (0.96–0.98)	39,656 (34.3%)	287,267 (34.9%)	0.98 (0.97–0.99)	0.96 (0.95–0.98)
High lockdown severity	31,690 (26.3%)	216,322 (25.0%)	1.05 (1.04–1.06)	1.08 (1.06–1.09)	31,308 (27.1%)	212,232 (25.8%)	1.05 (1.04–1.06)	1.08 (1.07–1.10)
iFOBT returned								
Low lockdown severity	14,698 (31.2%)	157,434 (45.7%)	0.99 (0.98–1.00)	0.98 (0.96–1.00)	10,214 (22.9%)	106,144 (33.0%)	0.98 (0.97–1.00)	0.97 (0.94–0.99)
Moderate lockdown severity	11,801 (28.3%)	128,295 (42.4%)	0.98 (0.96–0.99)	0.95 (0.93–0.97)	9286 (23.4%)	9569 (3.3%)	0.99 (0.97–1.01)	0.97 (0.95–0.99)
High lockdown severity	10,218 (32.3%)	103,698 (47.9%)	1.04 (1.03–1.06)	1.09 (1.06–1.11)	8068 (25.8%)	79,813 (37.6%)	1.03 (1.01–1.05)	1.07 (1.05–1.10)
Valid iFOBT								
Low lockdown severity	14,483 (30.7%)	155,949 (45.3%)	0.99 (0.98–1.00)	0.98 (0.96–1.00)	10,063 (22.6%)	105,277 (32.7%)	0.98 (0.97–1.00)	0.96 (0.94–0.99)
Moderate lockdown severity	11,597 (27.8%)	126,962 (41.9%)	0.97 (0.96–0.99)	0.95 (0.93–0.97)	9154 (23.1%)	94,870 (33.0%)	0.99 (0.98–1.01)	0.97 (0.95–1.00)
High lockdown severity	10,069 (31.8%)	102,749 (47.5%)	1.05 (1.03–1.06)	1.09 (1.06–1.11)	7957 (25.4%)	79,247 (37.3%)	1.03 (1.01–1.05)	1.07 (1.05–1.10)
Positive iFOBT								
Low lockdown severity	1566 (3.3%)	9746 (2.8%)	1.01 (0.97–1.06)	1.01 (0.95–1.08)	1122 (2.5%)	6812 (2.1%)	1.03 (0.98–1.08)	1.03 (0.96–1.11)
Moderate lockdown severity	1157 (2.8%)	7864 (2.6%)	0.93 (0.88–0.98)	0.90 (0.84–0.96)	935 (2.4%)	5985 (2.1%)	0.97 (0.92–1.03)	0.94 (0.88–1.02)
High lockdown severity	1059 (3.3%)	6273 (2.9%)	1.07 (1.01–1.13)	1.11 (1.04–1.18)	775 (2.5%)	4842 (2.3%)	1.00 (0.93–1.06)	1.03 (0.95–1.11)
Follow-up colonoscopy (after a positive iFOBT)								
Low lockdown severity	1002 (64.0%)	7466 (76.6%)	1.06 (1.01–1.11)	1.08 (1.00–1.17)	514 (45.8%)	4129 (60.6%)	1.09 (1.02–1.17)	1.14 (1.02–1.27)
Moderate lockdown severity	611 (52.8%)	5382 (68.4%)	0.90 (0.83–0.96)	0.86 (0.79–0.94)	360 (38.5%)	3394 (56.7%)	0.93 (0.85–1.02)	0.90 (0.80–1.01)
High lockdown severity	590 (55.7%)	4539 (72.4%)	1.03 (0.95–1.10)	1.06 (0.97–1.16)	328 (42.3%)	3104 (64.1%)	0.95 (0.87–1.05)	0.96 (0.85–1.08)

NBCSP: National Bowel Cancer Screening Program; NBCSP: National Bowel Cancer Screening Program; SMI: severe mental illness; IRR: incidence rate ratio; iFOBT: immunochemical faecal occult blood test.

The denominator for participants returning an iFOBT, returning a valid iFOBT and returning a positive iFOBT are the total numbers as represented by the rows describing the number invited to participate in the NBCSP. The denominator for participants who had a follow-up colonoscopy is represented by the rows describing returning a positive iFOBT.

a Low lockdown severity states (Northern Territory, Queensland, South Australia, Tasmania, Western Australia), Moderate lockdown severity states (New South Wales and Australian Capital Territory), high lockdown severity states (Victoria).

b Missing residential data for *n* = 970 (0.1%) and *n* = 1038 (0.1%) of before and during COVID samples, respectively.

c Adjusted for participant age, level of remoteness, index of socioeconomic disadvantage and sex.

## Discussion

The purpose of this study was to investigate the impact of COVID-19 on participation in Australia’s NBCSP, with a particular emphasis on people with SMI. We detected a 10% overall reduction in NBCSP participation from pre-COVID to during COVID. Compared to the modelling conducted by the Australian Government Department of Health at the onset of COVID (Nickson et al., 2023), our results suggest that the pandemic’s impact on NBCSP participation and CRC outcomes may be less severe than first predicted. Less than one-quarter (23.9%) of people with SMI participated in the NBCSP during the COVID-19 pandemic compared to 30.5% before. However, relatively speaking, people with SMI were less impacted by the pandemic than controls. Despite this, people with SMI remained less likely to return a valid iFOBT, and more likely to return a positive iFOBT result compared to people without SMI during the COVID pandemic, though these results were not statistically significant. They were also 13% less likely to have a follow-up colonoscopy after a positive iFOBT result. These findings underscore the need to provide targeted support for vulnerable groups to ensure equitable participation in the NBCSP and access to subsequent diagnostic and surgical procedures, even during times of global crisis.

COVID had a marked impact on NBCSP participation in Australia. Notably, there was a 10.3% overall reduction in participation, and a 13.5% overall reduction in the number of people who had a colonoscopy following positive iFOBT. The reduction in participation among the control cohort was more pronounced than it was for individuals with SMI (difference of 10.8% vs difference of 6.6%, respectively), although, the reduction in follow-up colonoscopy rates was greater among the SMI cohort compared to controls (difference of 15.8% vs difference of 13.1%, respectively). Ultimately, while NBCSP participation disparities between SMI and control groups lessened during COVID, disparities in follow-up colonoscopies worsened. Given how crucial colonoscopies are in early detection, precise staging and reducing CRC-related mortality (Brenner et al., 2016; Wang et al., 2024; Zhang et al., 2020), this is of concern.

Across Australia, there were temporary suspensions to CRC diagnostic and surgical procedures due to the pandemic. During 2020, Cancer Australia reported that there was a 13% reduction in CRC-related diagnostic procedures (amounting to 87,293 fewer service provisions), including colonoscopies and sigmoidoscopies with and without polyp removal (Cancer Australia, 2021). This was the greatest reduction observed among Australia’s top five incident cancers (including breast, colorectal, lung, prostate and skin cancers) (Cancer Australia, 2021). There was also a 1% reduction in surgical and non-surgical therapeutic procedures (e.g. resection of lesions, colectomy, hemicolectomy, abdominoperineal resections and anterior bowel resections) (Cancer Australia, 2021). Yet, this alone does not explain why the disparities in follow-up colonoscopies between people with and without SMI widened during the COVID pandemic. Nor do the differences in follow-up colonoscopies based on lockdown severity.

Across a range of health outcomes, Canadian researchers have described the impact of the pandemic on people with SMI as disproportionate and substantial (Kassam et al., 2023). Generally, people with SMI are less likely to engage in infection control practices, have reduced capacity for self-care and fewer coping resources (Kassam et al., 2023). COVID-19 then contributed additional challenges, such as rapidly changing infection control measures, social isolation, disruption to routine social and support services, and greater health anxiety (Kassam et al., 2023). Compounding this was a range of economic and social stressors such as unemployment and housing instability (Kaleveld et al., 2020; Kassam et al., 2023), which may have led to the deprioritisation of preventive healthcare measures. Moreover, COVID was considered a catalyst for the onset of new psychosis, as well as the exacerbation of existing symptoms for some people with SMI (Hamada and Fan, 2020). Undoubtedly, a combination of some or all of these factors could have contributed to reduced participation in Australia’s NBCSP, and particularly, reduced follow-up colonoscopies. People with SMI also face significant systemic barriers to accessing care in a more general sense, which may have contributed to the reduction we identified. Systemic barriers include stigma and discrimination associated with having a SMI, fragmentation between mental and physical health services, and care providers who lack the necessary skills and confidence to support them appropriately (Bull et al., 2024; Lawrence and Kisely, 2010). However, further investigation is required to understand the reality of the COVID-19 pandemic for Australians with SMI, as this remains unexplored. Without this information, we are ill-equipped to support this vulnerable population when the next pandemic arises.

It is noteworthy that the international literature has highlighted the impact of COVID-19 on the participation of other vulnerable groups across a range of screening programs. For example, in the United States, there were significant reductions to participation in the National Breast and Cervical Cancer Early Detection Program for vulnerable ethnic and racial groups during the first 6 months of the pandemic. Compared to White Americans, 4% fewer Black Americans, 25% fewer Asian/Pacific Islander Americans, 30% fewer American Indian/Alaskan Native Americans and 17% fewer Multiracial Americans received breast cancer screening (DeGroff et al., 2021). Similar patterns emerged for cervical cancer screening (DeGroff et al., 2021). A population-based retrospective study from Ontario, Canada also illustrated that older individuals, those living in lower income neighbourhoods, and First Nations people were significantly more likely to experience diagnostic delay after abnormal breast, cervical and CRC screening tests during the pandemic (Walker et al., 2021). These known disparities – linked to poverty, poor access to care and other social determinants of health (Wentzensen et al., 2021) – have been exacerbated irrevocably by COVID, and are now being realised in the forms of delayed diagnosis, upstaged cancers and higher likelihood of mortality (Nodora et al., 2021).

To address the significant disparities we identified in follow-up colonoscopies among people with SMI, three key public health recommendations should be considered. First, NBCSPs need to purposefully target vulnerable groups to raise awareness of the NBCSP, demonstrate its importance and ensure accurate information is being shared. In particular, increased awareness around the importance of undergoing colonoscopies following a positive iFOBT result is crucial. This will also serve to address the ‘infodemic’ (i.e. ‘*too much information including false or misleading information in digital and physical environments during a disease outbreak*’; World Health Organization, 2024) that will inevitably accompany the next global pandemic. Second, greater collaboration between mental health services and NBCSP providers needs to occur to integrate critical screening information and advice into the routine care structures of people with SMI. This should also include providing supportive environments where people with SMI can undertake their iFOBTs and follow-up colonoscopies, thereby reducing the access barriers this population face. Finally, the health inequities experienced by people with SMI in our analysis are not new. Greater financial investment in the aforementioned strategies, as well as strategies to reduce health inequities for people with SMI, are needed to reduce the short- and long-term consequences of CRC.

### Limitations

Despite the comprehensive dataset underpinning the current study, there are some limitations. First, the ‘during COVID’ dataset only comprised invitations to the NBCSP up until 31 July 2021. Resultantly, our examination did not span the entirety of the COVID-19 pandemic period, nor were we able to consider COVID-related CRC mortality. This is a critical area for future research. Second, we lacked information about covariates such as ethnicity, country of birth, marital status, educational attainment, comorbidities, social functioning and lifestyle factors such as diet, tobacco use, alcohol consumption and other substance use. Moreover, we were unable to include First Nations status in model adjustment due to significant missing data. The lacking information and inability to include First Nations status in our analyses could limit the generalisability of our findings, as these factors may also influence participation in the NBCSP and subsequent outcomes. Finally, Australia suffered several catastrophic weather events at the beginning of the COVID-19 pandemic. These included drought, severe bushfires, cyclones, hail storms and floods across New South Wales, Victoria and Queensland (ABS, 2020). These events caused huge displacement and upheaval to many Australians, and may have impacted on NBCSP participation.

## Conclusion

Findings of the current study underscore an urgent need for targeted public health initiatives that promote greater participation in the NBCSP, particularly follow-up colonoscopies, for people with SMI. Implementing targeted awareness strategies, promoting greater collaboration between mental health services and NBCSP providers, and investing in the reduction of health inequities are all crucial public health strategies that will bridge the gap in CRC outcomes between people with and without SMI. In turn, these efforts will reduce health disparities exacerbated by the COVID-19 pandemic, ensuring that vulnerable populations receive appropriate care and support in both the short and long term.
